# Toxicity, mutagenicity, and source identification of polycyclic aromatic hydrocarbons in ambient atmosphere and flue gas

**DOI:** 10.1007/s11356-024-35494-x

**Published:** 2024-11-15

**Authors:** Shih Yu Pan, Ya Syuan Wu, Yu-Cheng Chen, Yen-Shun Hsu, Yu Chi Lin, Pao Chen Hung, Charles C.-K. Chou, Somporn Chantara, Yuan Cheng Hsu, Kai Hsien Chi

**Affiliations:** 1 Institute of Environmental and Occupational Health Sciences, National Yang Ming Chiao Tung University, Taipei, 112 Taiwan; 2 National Institute of Environmental Health Sciences, National Health Research Institutes, 35 Keyan Road, Zhunan Town Miaoli, Taiwan; 3 School of Applied Meteorology, Nanjing University of Information Science & Technology, Nanjing, China; 4 Kyulien Environment Improving Co., Ltd., Taoyuan, 330 Taiwan; 5 Research Center for Environmental Changes, Academia Sinica, Taipei, 115 Taiwan; 6 Environmental Science Research Center, Faculty of Science, Chiang Mai University, Chiang Mai, Thailand; 7 National Environmental Research Academy, Ministry of Environment, Taoyuan, 330 Taiwan

**Keywords:** PM_2.5_, Polycyclic aromatic hydrocarbons, Power plants, Gasoline and diesel vehicles, Source apportionment, Lifetime excess cancer risk

## Abstract

This study aimed to assess the characteristics of particulate matter (PM) and polycyclic aromatic hydrocarbons (PAHs) from various stationary and mobile emission sources in Taiwan, with a focus on source apportionment and associated health risks. The northern power plant, equipped with bag filters operating at 150 °C, had significantly lower FPM and CPM levels (0.44 and 0.13 mg/m^3^, respectively) compared to the central and southern power plants, which used electrostatic precipitators operating at 250 °C (FPM, 1.45–8.35 mg/m^3^; CPM, 2.37–3.73 mg/m^3^). Additionally, emissions from diesel vehicles under both idle and high-speed conditions exhibited higher FPM levels (3.46–4.67 mg/m^3^) than gasoline vehicles (0.19–0.40 mg/m^3^). In terms of PAH toxicity, diesel vehicle emissions had significantly higher BaP-TEQ (87.3 ng/m^3^) and BaP-MEQ (25.9 ng/m^3^) levels compared to power plants (BaP-TEQ, 5.49 ng/m^3^; BaP-MEQ, 2.65 ng/m^3^). The highest ambient concentrations of PM2.5, BaP-TEQ, and BaP-MEQ were recorded at traffic sites, with values of 48 ± 36 µg/m^3^, 0.29 ng/m^3^, and 0.11 ng/m^3^, respectively. Differences in PAH distributions between stationary and mobile sources were influenced by factors such as pollution control technologies, combustion temperatures, and fuel types. Diesel vehicle emissions were dominated by benzo[g,h,i]perylene (BghiP), indeno[1,2,3-cd]pyrene (IND), benzo[a]pyrene (BaP), and benzo[b]fluoranthene (BbF) under idle conditions, while phenanthrene (PA), pyrene (Pyr), and BghiP were prevalent under high-speed conditions. Source apportionment conducted using principal component analysis (PCA) and positive matrix factorization (PMF) identified diesel and gasoline vehicles as the dominant contributors to atmospheric PAHs in Taiwan, accounting for 38% of the total, followed by coal-fired power plants at 35%. The highest lifetime excess cancer risk (ECR) of 2.5 × 10⁻^5^ was observed in traffic-dense areas, emphasizing the public health implications of vehicle emissions. The study adds credibility to the source apportionment findings, and the health risk analysis highlights variations across different regions, including traffic, urban, rural, and background zones.

**Supplementary Information:**

The online version contains supplementary material available at 10.1007/s11356-024-35494-x.

## Introduction

Polycyclic aromatic hydrocarbons (PAHs) are a class of organic compounds composed of two or more aromatic rings of carbon and hydrogen atoms. These compounds are produced through incomplete combustion of organic materials and are commonly emitted from various sources, including flue gas from industrial processes and vehicle exhaust (Napier et al. 2008). The combustion of coal for energy production, as well as emissions from gasoline and diesel vehicles, contributes significantly to the presence of PAHs in the atmosphere (Bi et al. 2020; Kermani et al. 2021; Slezakova et al. 2013; Sulong et al. 2019). The US Environmental Protection Agency (US EPA) has classified 16 PAHs as priority pollutants due to their carcinogenic properties, with 8 identified as mutagenic congeners by the International Agency for Research on Cancer (IARC) (International Agency for Research on Cancer 2011). Among these 16 PAHs, benzo[a]pyrene is categorized as carcinogenic to humans (group 1), while dibenz[a,h]anthracene is classified as probably carcinogenic to humans (group 2A) (Boström et al. 2002, Gammon &Santella 2008, Luch 2005, Okona-Mensah et al. 2005, Rybicki et al. 2006, Samburova et al. 2017). Generally, the toxicity concentration of PAHs (BaPeq) serves as a measure to assess the toxicity and carcinogenic in PAHs (Nisbet &Lagoy 1992).

In the atmosphere, PAHs could be bound to particulate matter (PM), particularly PM_2.5_, which poses a serious health risk due to its ability to penetrate deep into the lungs and respiratory tract. PM_2.5_ originates from both primary and secondary sources. Primary PM is directly emitted from stationary sources such as power plants, coal-fired boilers, and industrial facilities, as well as mobile sources like gasoline and diesel vehicles (Hsu et al. 2016; Liu et al. 2008; Shen et al. 2011; Slezakova et al. 2013; Wang et al. 2015; Zhu et al. 2019; Zielinska et al. 2004). Secondary PM is generated in the atmosphere through intricate chemical reactions involving gases and organic compounds (Bi et al. 2020; Brewer et al. 2016; Kermani et al. 2021; Querol et al. 2001; Yang et al. 2018). In the emission source, total particulate matter (TPM) including both condensable and filterable particulate matter (CPM and FPM) was measured and could be the indicator of some emission sources. In coal-fired boilers, levels of CPM were significantly higher than that of FPM (Wu et al. 2021). Emissions from coal-fired boilers contain 10–30 times more CPM than FPM, as demonstrated in a previous study (Ngo et al. 2022). Air pollution, primarily attributed to suspended fine PM, that is, PM with a diameter of ≤ 2.5 µm (PM_2.5_), poses severe health risks, including damage to the lungs and the respiratory tract, leading to millions of deaths worldwide annually (Kim et al. 2015). PM_2.5_ has absorbed and contains various chemical compounds, such as polycyclic aromatic hydrocarbons (PAHs), water-soluble ions, metal elements, or black carbon, some of which are toxic (Chen et al. 2016; Dockery et al. 1993; Hsiao et al. 2021; Liu et al. 2016; Wang et al. 2021).

In Taiwan, rapid economic growth and urbanization have led to increased vehicle numbers, particularly in congested urban areas such as Taipei (Hao et al. 2019; Lin et al. 2019b). Mobile sources, particularly diesel engines, are major contributors to air pollution, with particulate matter concentrations from diesel engines reported to range between 3880 and 16,800 μg/m^3^, significantly higher than those from gasoline engines, which range from 361 to 1020 μg/m^3^ (Lin et al. 2019a, 2019b). The PAH concentration in diesel exhaust emissions is also substantial, ranging between 28.4 and 43.4 ng/Nm^3^ (Lin et al. 2006, 2019b). Given the substantial contribution of mobile sources to PM_2.5_ and PAH pollution, individuals in urban areas are more likely to be exposed to vehicle emissions than stationary sources (Hsiao et al. 2021).

The growing concern over the detrimental health effects of exposure to chemical compounds has been underscored by recent research (Kim et al. 2015; Ngo et al. 2018, 2019; Sulong et al. 2019; Zhang et al. 2021). Numerous studies have investigated the health risks associated with PAHs, including assessments of incremental lifetime cancer risk (ILCR) and the lifetime excess cancer risk of PM_2.5_-bound PAHs (Boström et al. 2002; Famiyeh et al. 2021; Kumar et al. 2020; Slezakova et al. 2013; Yan et al. 2019). For example, a study evaluating the lifetime excess cancer risk (ECR) resulting from exposure to PAH emissions from industrial plants in central Taiwan reported a value of 4.7 × 10^−5^. This finding indicated that the ECR exceeded the recommended inhalation lifetime cancer risk range (10^−6^ to 10^−5^) set by the US EPA and the World Health Organization (WHO) (Albert 1994; Barlow et al. 2006; Chen et al. 2016).

A recent study found that the BaPeq concentration in emission particles from the stack of the Taiwan power plant was 479 ± 284 ng/mg, while coal-related stationary sources ranged from 303–360 ng/m^3^ (Ngo et al. 2022). Additionally, research indicates that gasoline emissions contain Σ21 PAHs with concentrations of 206 ± 42.4 ng/m^3^, whereas diesel emissions range from 1320–4230 μg/m^3^ (Lin et al. 2006, 2019a). Overall, mobile sources tend to have higher concentrations of PAHs compared to stationary sources. Previous studies have highlighted various health impacts associated with PAH exposure, including an increased risk of inhalation cancer, elevated levels of oxidative stress, ischemic heart disease, systemic inflammation, and potential effects on children’s cognitive and behavioral development (Kongpran et al. 2021; Liu et al. 2015, 2020; Yan et al. 2019).

Additionally, this study collected the PM_2.5_-bounded PAHs in different environments, such as traffic-heavy areas, urban regions, rural regions, and cleaner background zones. By analyzing source apportionment and excess cancer risk (ECR) separately for traffic, urban, rural, and background regions, this study fills a critical gap in understanding how PAH-related health risks vary across different geographic zones.

## Materials and methods

### Emission source and ambient sampling sites

In our study, the CPM and FPM were collected from the exhaust emitted by coal-fired power plants and mobile sources with gasoline and diesel engines. Additionally, PM_2.5_ samples were collected from fourteen ambient air sampling sites to assess pollution contributions from stationary and mobile sources, including coal-fired power plants and vehicle emissions (Fig. S1).

The characteristics of emission sources from power plants and vehicles, including gasoline and diesel vehicles under idle operating and high-speed (70 km/h) driving conditions, are summarized in Table S1. All power plants in Taiwan were equipped with air pollution control devices (APCDs), such as De-NOx, De-SOx, and dust removal equipment (Hsu et al. 2016). However, the systems and dust removal equipment used varied slightly among different power plants. The northern region’s power plants utilized selective catalytic reactors (SCR), seawater flue gas desulfurization (SWFGD) systems, and bag filters, while those in the central region employed electrostatic precipitators (ESPs), SCR, and limestone flue gas desulfurization systems. Gasoline and diesel vehicles included in the study were manufactured in 1997 and 2002, respectively, with engine displacements exceeding 2000 cm^3^. The samples from stack flue gas samples of power plants and vehicle emissions were analyzed to study the characteristic profiles.

Ambient air samples were collected from various regions of Taiwan from 2015 to 2019. The background sampling sites (background sample 1 [B1], *n* = 2) were situated at the Lulin Mountain Meteorological Station, free from local pollution sources in central Taiwan. Five additional urban sampling sites (urban samples 1–5 [U1–U5], *n* = 25) were positioned along roadsides and near the main roads in northern, central, and southern Taiwan. Furthermore, six rural sampling sites (rural samples 1–6 [R1–R6], *n* = 29) were located near riverside areas, away from residential zones. Finally, two traffic sampling sites (traffic samples 1–2 [T1 and T2], *n* = 10) were positioned adjacent to the expressway and within the Bagua Mountain tunnel, far from other sources of pollution. A total of 66 air samples were collected, and their details are listed in Table S2.

### Sampling instrumentation and chemical analysis

This study followed the Taiwan EPA method NIEA A212.11B, NIEA A214.71C, and NIEA A730.70C to collect stack flue gas samples, analyzing for PM_2.5_ including FPM, CPM, and PAHs, respectively. Flue gas sampling employed cyclone separators known for their stable and reliable features. For vehicle emissions, chassis dynamometers were utilized to maintain constant emission flow rates, with PM_2.5_ collected using a cyclone set from Apex Instruments Co. Pvt. Ltd., West Bengal, India.

Ambient air samples were collected using high-volume sampling instruments from Analitica HVS-PM2.5 (AMS Analitica, Pesaro, Italy) and SIBATA HV-1000R (SIBATA Scientific Technology Ltd., Saitama, Japan), with flow rates ranging from 500 to 1000 L/min. Each sample collected a volume exceeding 700 m^3^ over a 24–48 h sampling duration. Quartz fiber filters (AMS Analitica, 150 mm, Whatman, USA; SIBATA, 8 × 10 inches, Pall Corporation, NY, USA) served as the sampling media (Pan et al. 2022a). Before sampling, all filters underwent baking at 400 °C for 4 h to eliminate remaining organic debris. Before gravimetric analysis, the filters were stabilized for 48 h at a constant temperature of 24 ± 1 °C and a relative humidity of 40 ± 5% to ensure accuracy. The dichloromethane extract from PM_2.5_ samples underwent Soxhlet extraction for 8 h to isolate the contents of 16 PAHs. Subsequently, the extract was concentrated to 1 mL using a vacuum rotary evaporator (BÜCHI Labortechnik AG, Flawil, Switzerland). Prior to extraction and analysis, each PM_2.5_ sample was supplemented with internal standards (deuterated PAHs and internal PAHs, 50 μL of 50 pg/μL for each sample). The method detection limits (MDL) for the 16 PAHs ranged from 0.30 to 2.1 ng/mL, achieving recovery efficiencies between 72 and 95%.

The collected samples underwent pretreatment for comprehensive chemical analysis, targeting PAHs, water-soluble ions (WSIs), and metal elements. A gas chromatography–tandem mass spectrometer (TSQ 8000 Evo Triple Quadrupole, Thermo Fisher Scientific, Waltham, MA, USA), equipped with a DM-5MS column (60 m × 0.25 mm × 0.25 μm, Dikma Technol. Inc., Foothill Ranch, CA, USA), was employed to detect the presence of 16 PAHs, including naphthalene (Nap), acenaphthylene (AcPy), acenaphthene (Acp), fluorine (Flu), phenanthrene (PA), anthracene (Ant), fluoranthene (FL), pyrene (Pyr), benz[a]anthracene (BaA), chrysene (CHR), benzo[b]fluoranthene (BbF), benzo[k]fluoranthene (BkF), benzo[a]pyrene (BaP), benzo[g,h,i]perylene (BghiP), indeno[1,2,3-cd]pyrene (IND), and dibenz[a,h] anthracene (DBA), along with their congeners (Ngo et al. 2022; Pan et al. 2022b). Metal element analysis included aluminum (Al), iron (Fe), sodium (Na), magnesium (Mg), potassium (K), calcium (Ca), strontium (Sr), barium (Ba), titanium (Ti), manganese (Mn), cobalt (Co), nickel (Ni), copper (Cu), zinc (Zn), molybdenum (Mo), cadmium (Cd), tin (Sn), antimony (Sb), thallium (Tl), lead (Pb), vanadium (V), cadmium (Cr), arsenic (As), yttrium (Y), selenium (Se), zirconium (Zr), germanium (Ge), rubidium (Rb), cesium (Cs), gallium (Ga), lanthanum (La), cerium (Ce), praseodymium (Pr), neodymium (Nd), samarium (Sm), europium (Eu), gadolinium (Gd), terbium (Tb), dysprosium (Dy), holmium (Ho), erbium (Er), thulium (Tm), ytterbium (Yb), lutetium (Lu), hafnium (Hf), and uranium (U). Detection of up to 46 elements was facilitated using an inductively coupled plasma mass spectrometer (NexION 300X; PerkinElmer, USA) (Ngo et al. 2019).

### Quality control and assurance

The method detection limits (MDL) for 27 PAHs varied between 0.30 and 2.1 ng/mL, with recovery efficiency falling within the range of 72 to 95% (Pan et al. 2022b). Analysis of ten WSIs, including chloride (Cl^−^), nitrate (NO_3_^−^), nitrite (NO_2_^−^), phosphate (PO_4_^3−^), sulfate (SO_4_^2−^), sodium (Na^+^), ammonium (NH_4_^+^), potassium (K^+^), calcium (Ca^2+^), and magnesium (Mg^2+^), was conducted using a Dionex ICS-1000 (Thermo Fisher Scientific, Waltham, MA, USA), with the recovery rates spanning from 88 to 104%.

### Source apportionment

Apportioning PAHs facilitates the estimation of the relative contributions from different emission sources. This study employed principal component analysis (PCA) and positive matrix factorization (PMF) models, widely recognized methods for determining the apportionment of various PAHs in samples, to identify the probable sources of PAHs. PCA is a multivariate statistical technique that transforms a dataset into various components while accounting for variance within the dataset (Malm et al. 1986; Salim et al. 2019). Eigenvalues and eigenvectors were derived from the correlation matrix, and principal factors with eigenvalues > 0.7 were selected. PMF, on the other hand, is a multivariate factor analysis technique. The PMF model (version 5.0; 2014) was developed by the US EPA to discern and quantify sources of PAHs (Callén et al. 2014, Paatero &Tapper 1994). In brief, the receptor model was applied as follows:

1 $$X=GF+\text{E}$$

Here, *X* represents the matrix (*m* × *n*) of the *m* chemical congeners measured in *n* samples. *G* is the matrix (*n* × *p*) of the contributions of *p* potential sources to the *n* samples. *F* is the matrix (*m* × *p*) of the *m* chemical congener profiles for the *p* potential sources. *E* is defined as the residual matrix.

### Calculation of lifetime excess cancer risk (ECR) from PAHs

The lifetime excess cancer risk (ECR) was utilized as a metric to assess the health risks associated with atmospheric PAHs. The values of BaP-TEQ (carcinogenic equivalent, ng/m^3^) and BaP-MEQ (mutagenic equivalent, ng/m^3^) were computed by multiplying the concentrations of each PAH compound. Hence, the determination of toxic and mutagenic concentrations was performed using the following equations:

2 $$\Sigma BaP-TEQ={\sum }_{i=1}^{n}\left({C}_{i}\times {TEF}_{i}\right)$$

3 $$\Sigma BaP-MEQ={\sum }_{i=1}^{n}\left({C}_{i}\times {MEF}_{i}\right)$$

Here, C_i_ represents the concentration of the congener PAH (ng/m^3^), TEF_i_ is the toxic equivalency factor [TEF_*i*_, Eq. (2)], and MEF_*i*_ is the mutagenic equivalence factor specific to that PAH [MEF_*i*_, Eq. (3)] (Durant et al. 1996; Liu et al. 2018, Nisbet &Lagoy 1992).

These calculations enabled the assessment of the cancer potency of each PAH compound relative to BaP. The ECR values (Eq. 4) were estimated using BaP-TEQ and BaP-MEQ by multiplying the unit cancer risk factor (URBaP) relative to BaP (8.7 × 10^−5^ per ng/m^3^) (Callén et al. 2014; Chen et al. 2016; Kong et al. 2018; Yu et al. 2016). The resulting ECR indicated the cancer risk associated with PAH exposure at the residential site:

4 $$ECR={UR}_{BaP}\times BaP-TEQ or BaP-MEQ$$

According to the US EPA, ECR values ranging from 1 × 10^−6^ to 10^−5^ are considered tolerable, while values less than 1 × 10^−6^ are deemed negligible.

## Results and discussion

### The emissions characteristics of CPM-bound and FPM-bound PAHs in flue gas

**Fig. 1:**
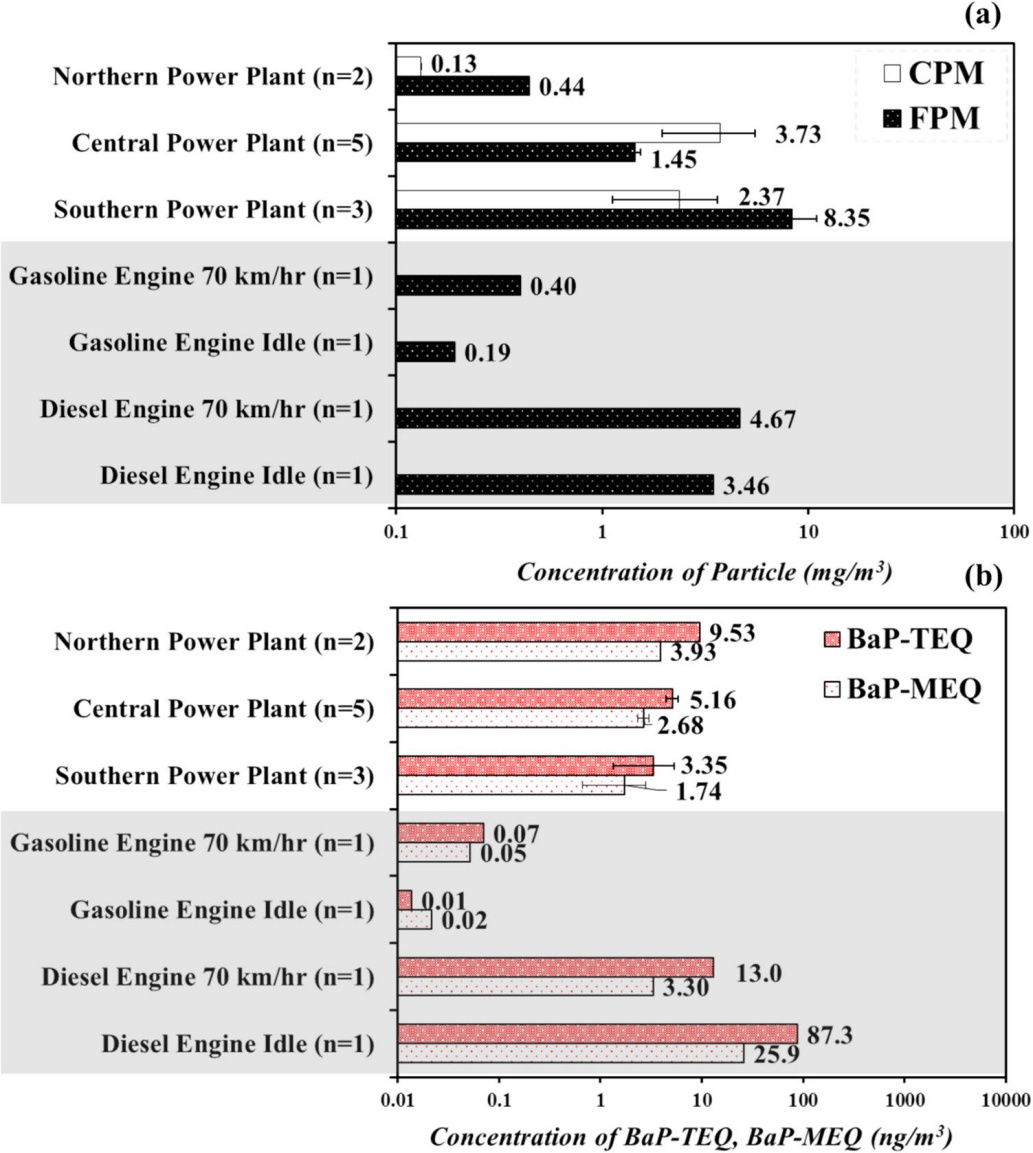
Concentration of **a** condensable particulate matters (CPMs) and filterable particulate matters (FPMs) and **b** BaP-TEQ and BaP-MEQ in FPM in different flue gases emitted from various sources

This study investigated PAHs bound to CPM and FPM emitted from power plants and vehicle emissions in Taiwan. Figure 1a illustrates the calculated concentrations of CPM and FPM, with the sample size indicated as *n*. Notably, among the power plants, the central power plant exhibited the highest CPM concentration (3.73 ± 1.77 mg/m^3^), while the southern power plant had the highest FPM concentration (8.35 ± 2.60 mg/m^3^). Conversely, the northern power plant, equipped with bag filters and operated at a lower temperature (150 °C) compared to the central and southern power plants (over 250 °C), displayed the lowest concentrations of both CPM and FPM. This variance in concentrations across power plants in different regions may be attributed to variations in the use of diverse APCDs and operating temperatures. Previous studies have indicated that thermal processes, often involving incomplete combustion, are the primary sources of most PAH emissions (Hsu et al. 2016; Pergal et al. 2013). For CPM, the removal effectiveness and flue gas temperature exhibited a highly significant association, implying that higher operation temperatures could increase CPM concentration (Wang et al. 2020). Despite utilizing the same APCDs, the southern power plant registered significantly higher FPM concentrations compared to the central power plant in this study, possibly due to operating under higher water vapor content (more than 35%) with lower removal efficacy in ESPs. This underscores the dominance of thermal processes in PAH emission.

The emissions from diesel vehicles operating at high speeds (70 km/h) exhibited higher FPM concentrations compared to gasoline vehicles under idle operating conditions, as depicted in Fig. 1a, owing to variations in fuel types and operating speeds without APCDs. The PM_2.5_ concentrations from diesel vehicle emissions (3.46–4.67 mg/m^3^) in this study aligned with those reported in a previous study (3.88–16.5 mg/m^3^) (Lin et al. 2019a). Furthermore, the PM_2.5_ concentrations from gasoline vehicle emissions (0.19–0.40 mg/m^3^) were lower compared to other studies (0.36–1.02 mg/m^3^) (Lin et al. 2019a, 2019b). Clearly, the PM_2.5_ concentration in mobile source emissions is influenced by fuel type and operating conditions, with diesel engines lacking exhaust gas treatment devices.

Figure 1b presents the concentrations of BaP-TEQ and BaP-MEQ from each emission source. The northern power plant exhibited higher concentrations of BaP-TEQ (9.53 ng/m^3^) and BaP-MEQ (3.93 ng/m^3^) compared to power plants in other regions. This discrepancy is likely due to differences in operating temperatures, which can impact PAH production (Hsu et al. 2016; Masclet et al. 1987). Additionally, the concentrations of Σ16 PAHs (25.6–66.2 ng/m^3^) in the power plant emissions were lower in this study than those reported in Anhui Province, China (Σ16 PAH concentration, 8840–22,580 ng/m^3^) (Wang et al. 2015), possibly due to variations in boiler capacities and APCDs with removal efficiency, ESP conditions, FGD, etc. Higher BaP-TEQ and BaP-MEQ concentrations were observed in diesel vehicle emissions at idle due to lower burning efficiency. Moreover, PAH concentrations in vehicle emissions in this study (BaP-TEQ values ranging from 0.01 to 87.3 ng/m^3^) were notably lower than those reported in Taiwan (PAH concentrations in diesel vehicle emissions, 5310 ng/m^3^; PAH concentrations in gasoline vehicle emissions, 206 ng/m^3^) (Lin et al. 2019a, 2019b).

**Fig. 2:**
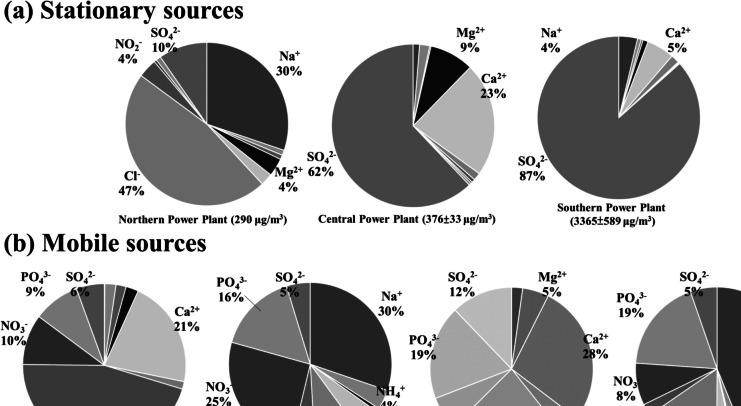
Distribution of water-soluble ions in FPM in different flue gases from various emission sources: **a** stationary sources and **b** mobile sources

**Fig. 3:**
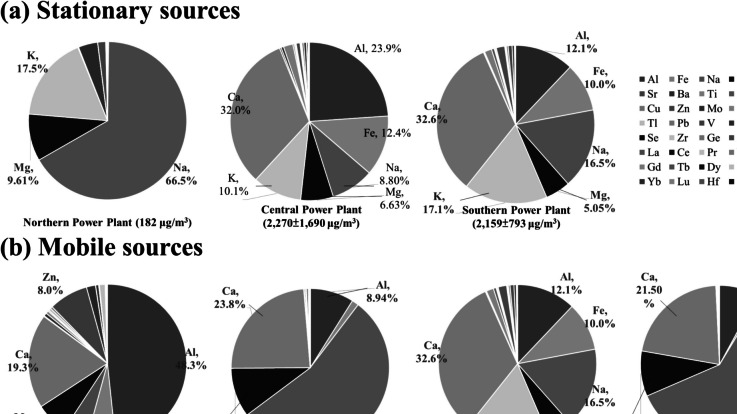
Distribution of metal compounds in FPM in different flue gases from various emission sources: **a** stationary sources and **b** mobile sources

The ions and metal compound distributions in each emission sample from the current study are illustrated in Figs. 2 and 3. The power plant in southern Taiwan exhibited elevated concentrations of water-soluble ions (3365 ± 589 μg/m^3^) compared to the power plants in other regions, likely due to operation at lower temperatures and under conditions of high water vapor content (Wang et al. 2019). Additionally, higher concentrations of SO_4_^2−^ were observed in the central and southern power plants compared to the northern power plant, attributable to the utilization of CaCO_3_ and varied APCDs, influencing the amount of SO_4_^2−^ released. Regarding vehicle emissions, the ion concentrations in emissions from diesel vehicles under idle operating conditions (168 μg/m^3^) surpassed those from other vehicles. Conversely, the ion concentrations in emissions from gasoline vehicles under high-speed driving conditions (26.4 μg/m^3^) were lower compared to diesel vehicles. Significant concentrations of NO_2_^−^ and Ca^2+^ were detected in emissions from vehicles under high-speed driving conditions, irrespective of engine type, and operating at high temperatures (Chiang et al. 2012). Conversely, significant concentrations of NO_3_^−^ and Na^+^ were observed in emissions from vehicles under idle operating conditions, regardless of engine type and lubricating oil (Fig. 2) (Alves et al. 2015; Habib 2017).

Figure 3 illustrates high concentrations of metal detected in emissions from the power plant in central Taiwan (2270 ± 1690 μg/m^3^) and emissions from diesel vehicles under idle operating conditions (752 μg/m^3^; Fig. 3). Al, Fe, and K were identified as the three predominant congeners in vehicle emissions, correlating with fuel combustion in the engine. This finding aligns with previous studies, such as Hao et al. (2019), indicating consistency in metal emission patterns from diesel engines. The quantity of PM in emissions is contingent upon factors such as fuel type and the efficacy of APCDs. These results underscore the composition and disparities between emissions from stationary and mobile sources, shedding light on the diverse nature of pollutant emissions.

### *The characteristics of PM*_*2.5*_*-bound PAHs in ambient air*

**Fig. 4:**
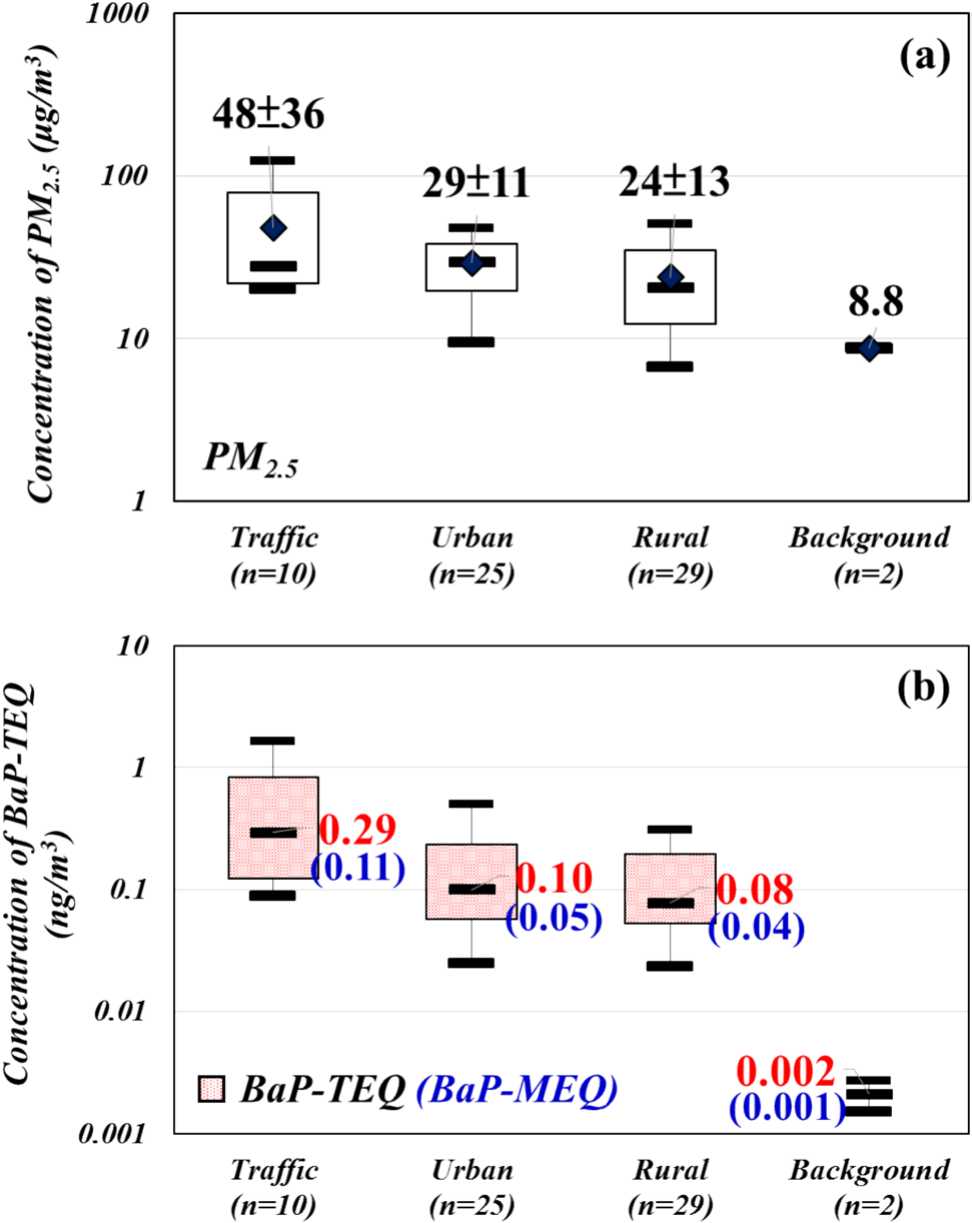
Ambient air concentration of **a** mass and **b** BaP-TEQ and BaP-MEQ in PM_2.5_ in different sampling areas

The average concentrations ranged from 8.75 to 72.2 μg/m^3^ for atmospheric PM_2.5_ and from 0.053 to 1.99 ng/m^3^ for atmospheric PAHs, respectively (Table S3). Notably, significantly higher concentrations of PM_2.5_ (72.2 ± 39.9 μg/m^3^) and BaP-TEQ (0.91 ± 0.46 ng/m^3^) were detected in the traffic samples compared to the other ambient air samples (Fig. 4). These findings are consistent with previous studies (Chen et al. 2016; Lin et al. 2019b). Importantly, none of the BaP-TEQ concentrations detected in this study exceeded the limits recommended in European guidelines (BaP-TEQ = 1.0 ng/m^3^; Directive 2004/107/EC, European Union). The PM_2.5_ concentrations in ambient air exhibited variation across different samples, with higher levels observed in the traffic samples ranging from 24.1 ± 3.43 to 72.2 ± 39.9 μg/m^3^, followed by 18.4 ± 6.20 to 38.0 ± 4.36 μg/m^3^ in urban samples, 14.7 ± 6.50 to 38.0 ± 8.97 μg/m^3^ in rural samples, and 8.75 μg/m^3^ in the background sample, as shown in Fig. 4a. These measurements align with previous findings in Taipei City (PM_2.5_ concentrations ranged from 10.5 ± 3.60–30.0 ± 8.50) (Hsiao et al. 2021) and in Changhua City (PM_2.5_ concentration ranged from 28.4 ± 17.5–35.0 ± 22.3) (Chen et al. 2016), indicating the influence of vehicle emissions on ambient PM_2.5_ concentrations (Chen et al. 2016; Hsiao et al. 2021; Kermani et al. 2021; Pfeffer 1994; Zhang et al. 2017).

The concentrations of BaP-TEQ ranged from 0.12 ± 0.03 to 0.91 ± 0.46 ng/m^3^ in the traffic samples, 0.05 to 0.30 ± 0.13 ng/m^3^ in the urban samples, 0.06 to 0.16 ± 0.11 ng/m^3^ in the rural samples, and 0.002 ng/m^3^ in the background sample. Similarly, the concentrations of BaP-MEQ ranged from 0.05 ± 0.01 to 0.33 ± 0.17 ng/m^3^ in the traffic samples, 0.03 ± 0.01 to 0.14 ± 0.05 ng/m^3^ in the urban samples, 0.02 ± 0.01 to 0.07 ± 0.04 ng/m^3^ in the rural samples, and 0.001 ng/m^3^ in the background sample (Table S3). Moreover, BaP-TEQ, BaP-MEQ, and PM_2.5_ concentrations exhibited similar trends (Fig. 4b). Compared to other urban environments in our study, higher PAH concentrations were detected in Taichung, Taiwan (average Σ21 PAH concentration, 56.1 ng/m^3^) (Fang et al. 2006), and Kuala Lumpur, Malaysia (average Σ16 PAH concentrations, 2.79 ng/m^3^) (Khan et al. 2015), whereas the concentrations of BaP-TEQ in the urban samples of this study were similar to observed in New York, USA (Σ8 BaP-TEQ concentration, 0.450 ng/m^3^; Σ8 BaP-MEQ concentration, 0.528 ng/m^3^) (Jung et al. 2010), but lower than those Mae Sot District, Thailand (Σ8 BaP-TEQ concentration, 0.41–3.14 ng/m^3^; Σ8 BaP-MEQ concentration, 0.34–3.35 ng/m^3^) (Janta et al. 2020). Previous studies have highlighted that the highest concentrations of BaP-TEQ and BaP-MEQ in traffic and urban sites surpass those in rural areas due to emissions from stationary and mobile sources, including vehicles and coal combustion.

### *The distribution and congener profile of PM*_*2.5*_*-bound PAHs in the emission sources and ambient air*

**Fig. 5:**
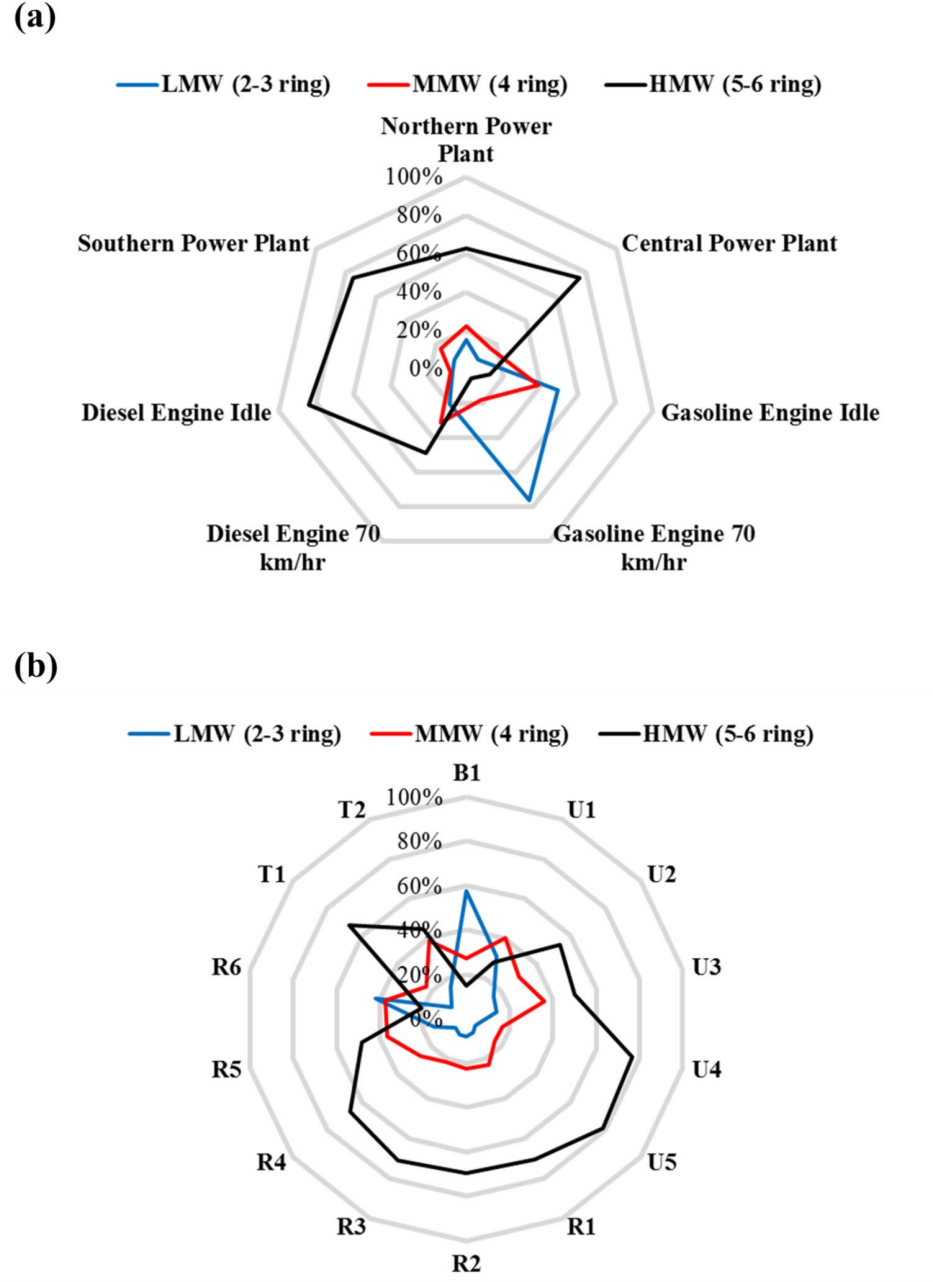
Distribution of low, medium, and high molecular weight PAHs in PM_2.5_ in different **a** flue gases emitted from emission sources and **b** ambient air sampling sites

The distribution of the PAHs and their congeners detected in flue gases from ambient air, stationary, and mobile sources is illustrated in Fig. 5. PAHs are composed of several aromatic rings, categorized based on the number of rings into low (2 or 3 rings), medium (4 rings), and high (5 or 6 rings) molecular weight PAHs. The proportions of high, medium, and low molecular weight PAHs in the stationary emission source ranged from 62.7–75.6%, 16.8–22.1%, and 7.55–15.1%, respectively (Fig. 5a). Predominant PAH species, such as BghiP, IND, and BbF, were found in the stationary emission sources, constituting proportions of 18.9–33.8%, 14.5–20.1%, and 7.07–12.7%, respectively. Previous studies have reported the presence of BghiP and BbF in power plant emissions (Kong et al. 2013). In contrast, the proportions of high, medium, and low molecular weight PAHs in the emissions from the diesel vehicles under both idle operating and high-speed driving conditions ranged from 48.7–84.2%, 8.31–31.2%, and 7.44–20.1%, respectively. However, the predominant PAH species differed between idle operating and high-speed driving conditions. Under idle conditions, BghiP (22.4%), IND (21.8%), BaP (17.4%), and BbF (10.9%) were predominant, while under high-speed conditions, PA (14.5%), Pyr (13.3%), and BghiP (10.6%) were dominant. Similarly, PAH species detected in emissions from the gasoline vehicles differed from those in diesel vehicle emissions. Low molecular weight PAHs constituted the majority of the PAHs in gasoline vehicle emissions, with predominant species including PA (37.3–55.9%), Pyr (8.07–19.3%), FL (9.10–15.5%), and Flu (8.23–17.0%), regardless of operating speed conditions. This distribution aligns with findings from another study where PAHs in emissions from gasoline vehicles mainly comprised 2 or 3 rings. Overall, the variation in PAH distribution in stationary and mobile sources can be attributed to differences in APCDs, burning temperatures, and types of fuels. Predominant PAH species such as BghiP, IND, and BbF were characteristic of stationary emission sources, while the major PAH species in diesel vehicle emissions under idle conditions were BghiP, IND, BaP, and BbF and under high-speed conditions were PA, Pyr, and BghiP. Gasoline vehicle emissions predominantly contained low molecular weight PAHs, with prevalent species such as PA, Pyr, FL, and Flu.

The proportions of PAHs in the ambient air samples categorized by molecular weight are presented in Fig. 5b. Low molecular weight PAH species were identified in the background sample and one of the rural samples (R6). Notably, the urban, rural, and traffic samples detected PAH species with high TEF values, such as BaP, BaA, BbF, and IND. These findings suggest that various human activities, including vehicle emissions and coal combustion, release toxic PAHs into the environment. Predominant PAH species, including FL, Pyr, PA, and NaP, were prevalent in this study’s urban, rural, and traffic samples. Similarly, some of these dominant species (Pyr and PA) were also found in diesel and gasoline vehicle emissions in previous studies (Alsberg et al. 1989; Harrison et al. 1996; Jang et al. 2013, Larsen &Baker 2003). Moreover, NaP serves as a tracer for petroleum emissions (Khairy &Lohmann 2013, Wang et al. 2014), while FL and Pyr are tracers for gasoline vehicle emissions (Ravindra et al. 2008). BghiP and IND are identified as tracers for petrochemical combustion and vehicle emissions in other studies (Dachs &Eisenreich 2000, Harrison et al. 1996; Simcik et al. 1999). In summary, the prevalence of species like FL, Pyr, PA, and NaP in urban, rural, and traffic samples suggests contributions from both vehicle emissions and coal combustion activities.

### Source apportionment of PAHs in emission sources and ambient air

In this study, we aimed to elucidate potential contribution sources using both flue gases and ambient air samples. However, the composition of atmospheric PAHs was influenced by multiple factors, leading to inconsistent results between PCA and PMF analyses. According to the PCA, PAHs primarily originated from vehicle emissions, whereas PMF analysis indicated that PAHs mainly stemmed from coal-fired power plant emissions (34.8%). Nonetheless, possible contributions were also attributed to gasoline (25.9%) and diesel (11.5%) vehicle emissions. Hence, there was a disparity in the results obtained from PCA and PMF analyses.

**Fig. 6:**
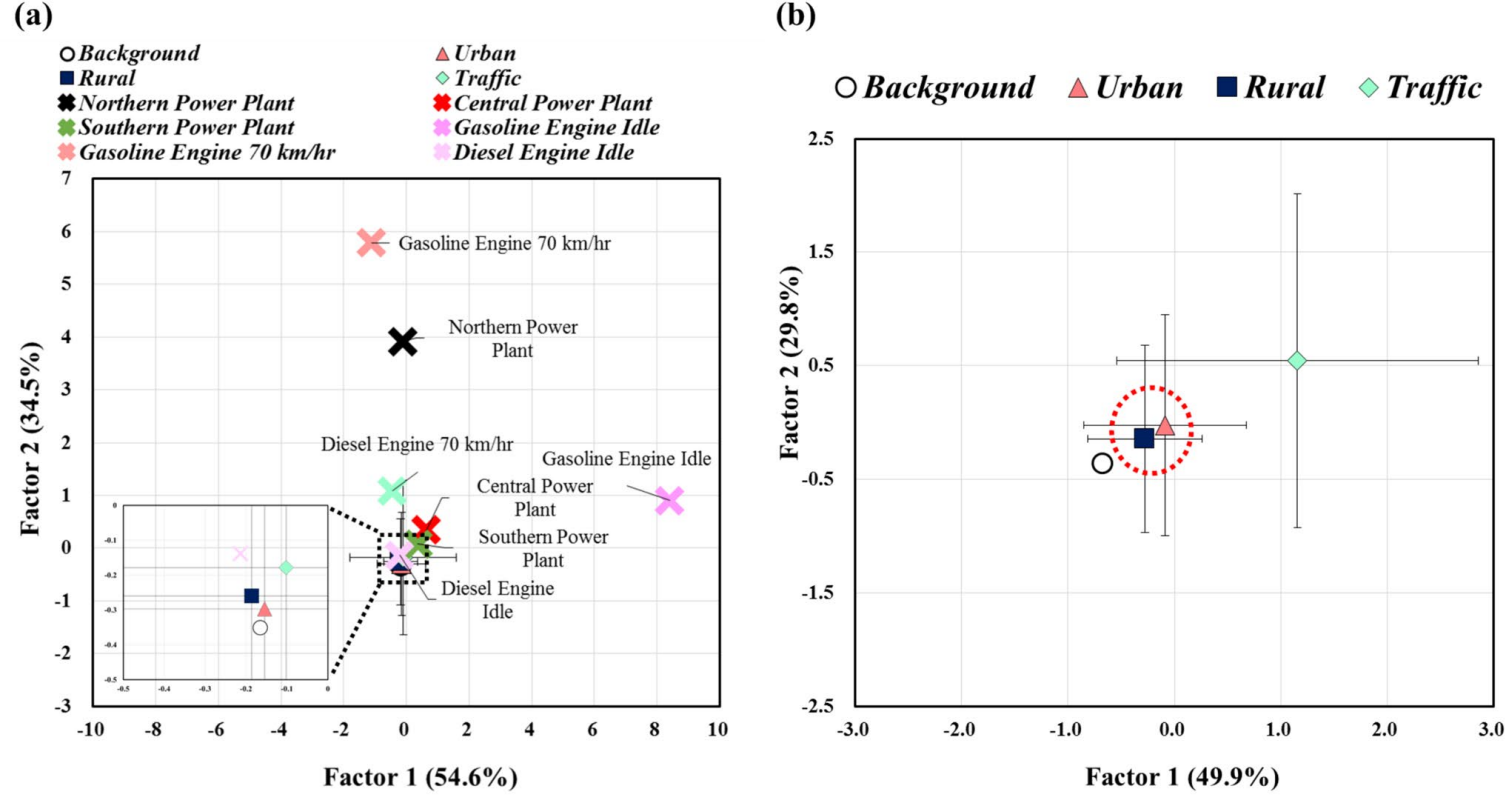
Score plot of PCA in **a** different flue gases (*n* = 14) with ambient air samples (*n* = 66) and **b** ambient air samples (*n* = 66) only

PCA is a commonly used method to identify sources in ambient air studies (Harrison et al. 1996, Larsen &Baker 2003, Malm et al. 1986, Salim et al. 2019). To ensure adherence to PCA standards, the varimax method with a rotation factor loading > 0.7 was employed for factor analysis. In this study, applying this method resulted in two major principal components: principal component 1 (PC1) explains 54.6% and principal component 2 (PC2) explains 34.5% of the total variance in the emission sources and ambient air samples (Fig. 6a). High molecular weight PAHs (PA, BaA, BbF, BkF, BaP, IND, DBA, and BghiP) were predominant in PC1, while low and medium molecular weight PAHs (Flu, Flt, and Pyr) were predominant in PC2 (Table S4(a)). These findings suggested that emission sources highly resemble those of power plants in central and southern Taiwan and diesel vehicles under idle operating conditions. Previous studies have also indicated significant contributions of PAHs from traffic and coal-burning sources (Brown &Brown 2012, Jang et al. 2013). According to the PCA results, the main PAHs in ambient air could be attributed to stationary sources such as power plants and mobile sources such as vehicles, as indicated by the differences observed in traffic, urban, rural, and background ambient air samples (Fig. 6b). When considering the PAH in different regions using PCA, PC1 and PC2 explained variances of 49.9% and 29.8%, respectively. The major PAH species found in PC1 were 5- or 6-ring PAHs, including NaP, PA, Ant, BaA, Chr, BbF, BkF, BaP, IND, and BghiP, suggesting emission sources from the power plant in central Taiwan, diesel vehicles under idle operating conditions, and gasoline vehicles under high-speed driving conditions. On the other hand, PAH species found in PC2 were 2–4-ring PAHs (NaP, Flu, PA, FA, and Pyr) (Table S4(b)), indicating similarities between urban and rural samples, likely influenced by the same emission sources (Fig. 6b). These PCA results are consistent with findings from other studies (Xiao et al. 2014) and support the idea that PAHs in ambient air originate from both stationary sources (e.g., power plants) and mobile sources (e.g., vehicles).

The source profiles generated by the PMF model are illustrated in Fig. S2, with the corresponding PAH species analyzed across various factors (Fig. S2(a)). These factors represent potential emission sources, including long-range transport (LRT) events, gasoline vehicles, coal combustion power plants, and diesel vehicles, associated with PAH percentages of 27.7%, 25.9%, 34.8%, and 11.5%, respectively (Fig. S2(b)). LRT events (*r* = 0.77) were found to be associated with PAH congeners such as FL and Pyr. These two congeners align with a previous study and were detected during LRT events in the northern region of Taiwan (Hsu et al. 2019). Gasoline vehicles (*r* = 0.53) were linked to PAH congeners NaP, PA, and FL, consistent with findings reported in another study. Notably, these PAH congeners detected in gasoline vehicle emissions were observed under both idle operating and high-speed driving conditions, as well as in air samples. Coal combustion power plants (*r* = 0.85) were associated with PAH congeners FL, Pyr, IND, and BghiP, which are recognized as source tracers of coal combustion in previous studies (Dat &Chang 2017, You 2008). Finally, diesel vehicles (*r* = 0.23) were linked to PAH congeners AcPy and Pyr, consistent with findings from other studies (Alsberg et al. 1989, Jiao et al. 2017, Larsen &Baker 2003, Varea et al. 2011). In summary, PMF analysis identified four potential emission sources contributing to PAH concentrations: long-range transport (LRT) events (27.7%), gasoline vehicles (25.9%), coal combustion power plants (34.8%), and diesel vehicles (11.5%).

### Risk assessment of ECR from PAHs

**Fig. 7:**
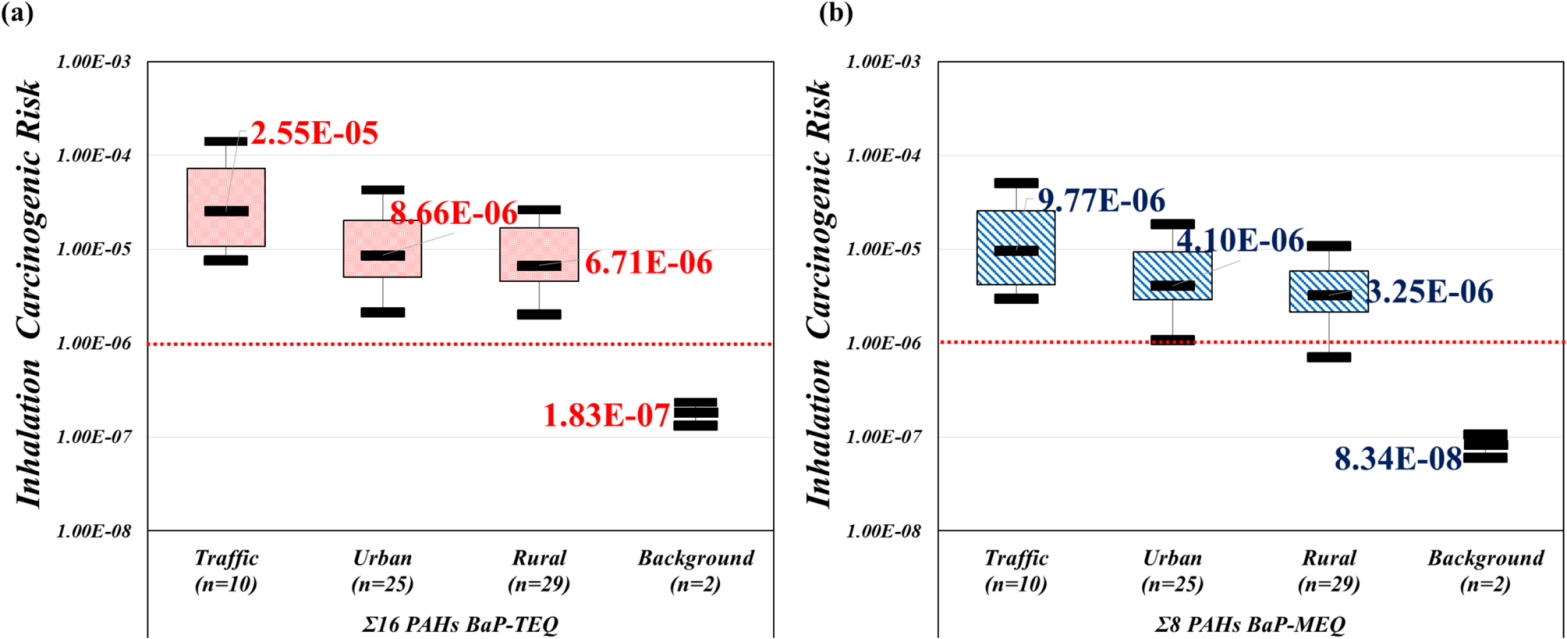
ECR was calculated using **a** BaP-TEQ and **b** BaP-MEQ concentrations in PM_2.5_ from different ambient air sampling sites

The ECR from atmospheric PAHs was calculated using the concentrations of Σ16 BaP-TEQ and Σ8 BaP-MEQ. In this study, the traffic, urban, rural, and background samples were associated with ECR values for BaP-TEQ of 2.55 × 10^−5^, 8.66 × 10^−6^, 6.71 × 10^−6^, and 1.83 × 10^−7^, respectively, and for BaP-MEQ of 9.77 × 10^−6^, 4.10 × 10^−6^, 3.25 × 10^−6^, and 8.34 × 10^−8^, respectively, as shown in Fig. 7. Comparable lifetime ECR values from Σ22 PAHs in northern and central Taiwan were reported to be 6.40 × 10^−5^ and 4.70 × 10^−5^, respectively (Chen et al. 2016; Hsu et al. 2019). The ECR values obtained in this study, ranging from BaP-TEQ of 1.83 × 10^−7^ to 2.55 × 10^−5^, align with previous findings. Several studies have identified vehicle emissions as the primary source of air pollutants in Taiwan (Chen et al. 2016; Fang et al. 2004; Lai et al. 2017; Lee et al. 1995; Ngo et al. 2022). Consistent with these findings, the traffic samples in our study exhibited the highest ECR in both BaP-TEQ (2.55 × 10^−5^) and BaP-MEQ (9.77 × 10^−6^) (Fig. 7), although these risks remain within tolerable ranges according to US EPA standards (Aminiyan et al. 2018; Santos et al. 2018). Combining spatial variation and source apportionment analyses, we identified vehicle emissions to be the primary pollutant source in Taiwan’s ambient air. Furthermore, by integrating potential sources and risk assessments, we apportioned the contribution of different pollution sources. The PMF analysis revealed coal combustion power plants (34.8%) as the most significant contributor, followed by long-range transport events (27.7%), with gasoline and diesel contributing over 37.0%. Meanwhile, the average ECR of BaP-TEQ (4.48 × 10^−5^) and BaP-MEQ (1.64 × 10^−5^) was highest in traffic sapling sites, mainly due to high TEQ congeners. Conversely, the lowest average ECR of BaP-TEQ (1.83 × 10^−7^) and BaP-MEQ (8.34 × 10^−8^) was found in the background sampling sites due to the absence of anthropogenic activities. Figure S3 displays the ECR of each contributor. Furthermore, we utilized the absolute contributions (concentrations, ng/m^3^) from PMF to calculate the excess lifetime cancer risk (Table S5). However, in several potential emission sources (long-range transport, gasoline vehicles, and coal combustion power plants), the ECR value exceeded 1 × 10^−6^, indicating these sources contributed more than 25.0% in the PMF model (Fig. S4). Therefore, cancer risk assessment suggests these sources pose significant health risks. Overall, our study offers valuable insights into the contribution of different pollution sources to exceed cancer risk, highlighting the importance of targeted mitigation strategies to minimize health impacts associated with PAH exposure.

## Conclusions

Our study observed higher concentrations of CPM in power plants adopted with high-temperature operated ESPs. These differences in emission levels were attributed to variations in APCD types and operating temperatures. Additionally, significant metal concentrations were detected from both power plants (182–2270 μg/m^3^) and diesel vehicles (709–752 μg/m^3^), with Al, Fe, and K being predominant. PM concentration in emissions varies depending on fuel type, APCDs utilized, etc. Apparently, higher concentrations of NO_2-_ and SO_4_^2−^ were attributed to the SWFGD system in the power plants, while the more significant proportion of Ca^2+^ and Na^+^ was linked to the use of CaCO_3_ in the central and southern power plants. The chemical composition varied due to differences in APCDs among these facilities.

The concentrations of BaP-TEQ varied across different samples, with traffic samples exhibiting higher levels (ranging from 0.12 ± 0.03 to 0.91 ± 0.46 ng/m^3^) compared to urban, rural, and background samples. This underscores the contribution of both stationary (e.g., power plants) and mobile sources (e.g., vehicles) to ambient air PAHs. The distribution of PAHs between stationary and mobile sources differed due to various factors such as APCD types, burning temperatures, and fuel types. Our study emphasizes vehicle emissions’ significant influence on atmospheric PM and PAH concentrations. We utilized multiple analytical approaches to consider various factors in source apportionment, identifying vehicles (with gasoline contributing 25.9% and diesel 11.5%) and coal burning (34.8%) as the primary sources of atmospheric PAHs. Overall, the significant ECR was attributed to PM_2.5_-bound PAHs from diesel engine vehicles.

## Supplementary Information

Below is the link to the electronic supplementary material.

Supplementary file1 (DOCX 3090 KB)

## Data Availability

Supporting data is provided in the manuscript.

## Abbreviations

FPM: Filterable particulate matter
CPM: Condensable particulate matter
APCD: Air pollution control devices
SCR: Selective catalytic reactors
SWFGD: Seawater flue gas desulfurization systems
ESPs: Electrostatic precipitators
PM: Particulate matter
PAHs: Polycyclic aromatic hydrocarbons
Nap: Naphthalene
AcPy: Acenaphthylene
Acp: Acenaphthene
Flu: Fluorine
PA: Phenanthrene
Ant: Anthracene
FL: Fluoranthene
Pyr: Pyrene
BaA: Benz[a]anthracene
CHR: Chrysene
BbF: Benzo[b]fluoranthene
BkF: Benzo[k]fluoranthene
BaP: Benzo[a]pyrene
BghiP: Benzo[g,h,i]perylene
IND: Indeno[1,2,3-cd]pyrene
DBA: Dibenz[a,h] anthracene
LMW: Low molecular weight
MMW: Median molecular weight
HMW: High molecular weight
PCA: Principal component analysis
PMF: Positive matrix factorization
ECR: Lifetime excess cancer risk
