# Supplementary material for: Toxicity, mutagenicity, and source identification of polycyclic aromatic hydrocarbons in ambient atmosphere and flue gas
Source: Environ Sci Pollut Res Int. 2024 Nov 15;31(56):64688–702. doi: 10.1007/s11356-024-35494-x (PMC11624214; doi:10.1007/s11356-024-35494-x)
Supplement: Supplementary file 1 — Supplementary file1 (DOCX 3090 KB) [file 11356_2024_35494_MOESM1_ESM.docx]

**Toxicity, Mutagenicity, and Source Identification of Polycyclic Aromatic Hydrocarbons of Emitted and Atmospheric Fine Particulate Matter in Taiwan**

Shih Yu Pan^1^, Ya Syuan Wu^1^, Yu-Cheng Chen^2^, Yen-Shun Hsu^1^, Yu Chi Lin^3^,

Pao Chen Hung^4^, Charles C.-K. Chou^5^, Somporn Chantara^6^,

Yuan Cheng Hsu^7^, Kai Hsien Chi^1*^

*^1^Institute of Environmental and Occupational Health Sciences, National Yang Ming Chiao Tung University, Taipei 112, Taiwan.*

*^2^National Institute of Environmental Health Sciences, National Health Research Institutes, 35 Keyan Road, Zhunan Town, Miaoli, Taiwan.*

*^3^School of Applied Meteorology, Nanjing University of Information Science & Technology, Nanjing, China.*

*^4^Kyulien Environment Improving Co., Ltd., Taoyuan 330, Taiwan.*

*^5^Research Center for Environmental Changes, Academia Sinica, Taipei 115, Taiwan.*

*^6^Environmental Science Research Center, Faculty of Science, Chiang Mai University, Chiang Mai, Thailand.*

*^7^National Environmental Research Academy, Ministry of Environment, Taoyuan, 330, Taiwan.*

***Corresponding authors E-mail: khchi@nycu.edu.tw**

Table S1. The characteristics of stationary and mobile sources in Taiwan.

| Sources | | Location | Characteristics |
| --- | --- | --- | --- |
| Stationary Sources | Northern Power Plant | New Taipei | Coal fire power plant with selective catalytic reactors (SCR), seawater flue gas desulfurization (SWFGD), and bag filter. |
|  | Central Power Plant | Taichung | Coal fire power plant with SCR, ESP, and limestone FGD. |
|  | Southern Power Plant | Yunlin | Coal fire power plant with selective SCR, ESP, and SWFGD. |
| Mobile Sources | Gasoline Engine  (70 km/hr) | － | Japanese  Production year: 1997.  Vehicle age more than 5 years.  Engine displacement: 2,000 c.c. |
|  | Gasoline Engine Idle |  |  |
|  | Diesel Engine  (70 km/hr) |  | Japanese  Production year: 2002.  Vehicle age more than 5 years.  .Engine displacement: 2,835 c.c. |
|  | Diesel Engine Idle |  |  |

Table S2. The characteristics of ambient air sampling sites in Taiwan.

| Sampling sites | | Location | Sampling Date | Characteristics & Weather Conditions |
| --- | --- | --- | --- | --- |
| *Background* | B1 | Nantou | Nov., 2019 | Atmospheric meteorological station, high-altitude sampling site, 2,862 m above mean sea level and free from local pollution; |
| *Urban* | U1 | Taipei | Jan., 2019 | Atmospheric monitoring station in elementary school of Environmental Protection Agency, located at Taipei city and nearby the main road. |
|  | U2 | Changhua | Mar., 2018 | Air PM_2.5_ sampling site in high school and near by the Zhangbin industrial park. |
|  | U3 | Changhua | Mar., 2018 | Air PM_2.5_ sampling site in the community center |
|  | U4 | Taichung | Sep., 2015 | PM_2.5_ sampling site in elementary school nearby the Taichung main road. |
|  | U5 | Taichung | Apr., 2015 | PM_2.5_ sampling site in junior high school nearby the Taichung main road. |
| *Rural* | R1 | Miaoli | Sep., 2015 | Air PM_2.5_ sampling site of upwind and downwind in Da-an river. |
|  | R2 | Taichung | Mar., 2015 | Air PM_2.5_ sampling site of upwind and downwind in Dajia river. |
|  | R3 | Taichung | Aug., 2015 | Air PM_2.5_ sampling site of upwind and downwind in Wu river. |
|  | R4 | Changhua | Apr., 2015 | Air PM_2.5_ sampling site of upwind and downwind in Jhuoshuei river. |
|  | R5 | Taichung | Mar., 2018 | PM_2.5_ sampling site nearby industrial area in Taichung city. |
|  | R6 | New Taipei | Jan., 2019 | PM_2.5_ sampling site of university in the Yang-Ming mountain. |
| *Traffic* | T1 | Changhua | May, 2015  Sep., 2015 | PM_2.5_ sampling site nearby the expressway. |
|  | T2 | Changhua | May, 2015  Sep., 2015 | Tunnel PM_2.5_ sampling site in the Bagua mountain. |

Table S3. Concentration (average ± standard deviation) of PM_2.5_, Σ16 PAHs, Σ16BaP-TEQ and Σ8BaP-MEQ in ambient air sampling sites.

| Sampling sites | | PM_2.5_  (μg/m^3^) | Σ16 PAHs Con.  (ng/m^3^) | Σ16BaP-TEQ  (ng/m^3^) | Σ8BaP-MEQ  (ng/m^3^) |
| --- | --- | --- | --- | --- | --- |
| *Background* | B1 (n=2) | 8.75 | 0.053 | 0.002 | 0.001 |
| *Urban* | U1 (n=7) | 18.4±6.21 | 1.03±0.57 | 0.09±0.07 | 0.03±0.01 |
|  | U2 (n=2) | 25.9 | 0.85 | 0.05 | 0.04 |
|  | U3 (n=6) | 38.0±4.36 | 0.66±0.30 | 0.07±0.01 | 0.04±0.01 |
|  | U4 (n=5) | 32.8±14.0 | 0.93±0.26 | 0.30±0.13 | 0.14±0.05 |
|  | U5 (n=5) | 31.2±12.0 | 0.88±0.42 | 0.21±0.07 | 0.11±0.04 |
| *Rural* | R1 (n=5) | 24.5±11.5 | 0.95±0.51 | 0.14±0.08 | 0.05±0.03 |
|  | R2 (n=5) | 19.0±15.7 | 0.82±0.05 | 0.16±0.11 | 0.07±0.04 |
|  | R3 (n=5) | 22.0±14.1 | 0.88±0.20 | 0.14±0.07 | 0.06±0.02 |
|  | R4 (n=5) | 38.0±8.97 | 1.99±0.97 | 0.10±0.06 | 0.04±0.02 |
|  | R5 (n=2) | 34.1 | 1.53 | 0.06 | 0.04 |
|  | R6 (n=7) | 14.7±6.46 | 0.78±0.34 | 0.06±0.07 | 0.02±0.01 |
| *Traffic* | T1 (n=5) | 24.1±3.43 | 0.77±0.22 | 0.12±0.03 | 0.05±0.01 |
|  | T2 (n=5) | 72.2±39.9 | 6.28±3.12 | 0.91±0.46 | 0.33±0.17 |

Table S4. Factor loading in 16 species of PCA. (a) Emission sources and ambient air (b) ambient air.


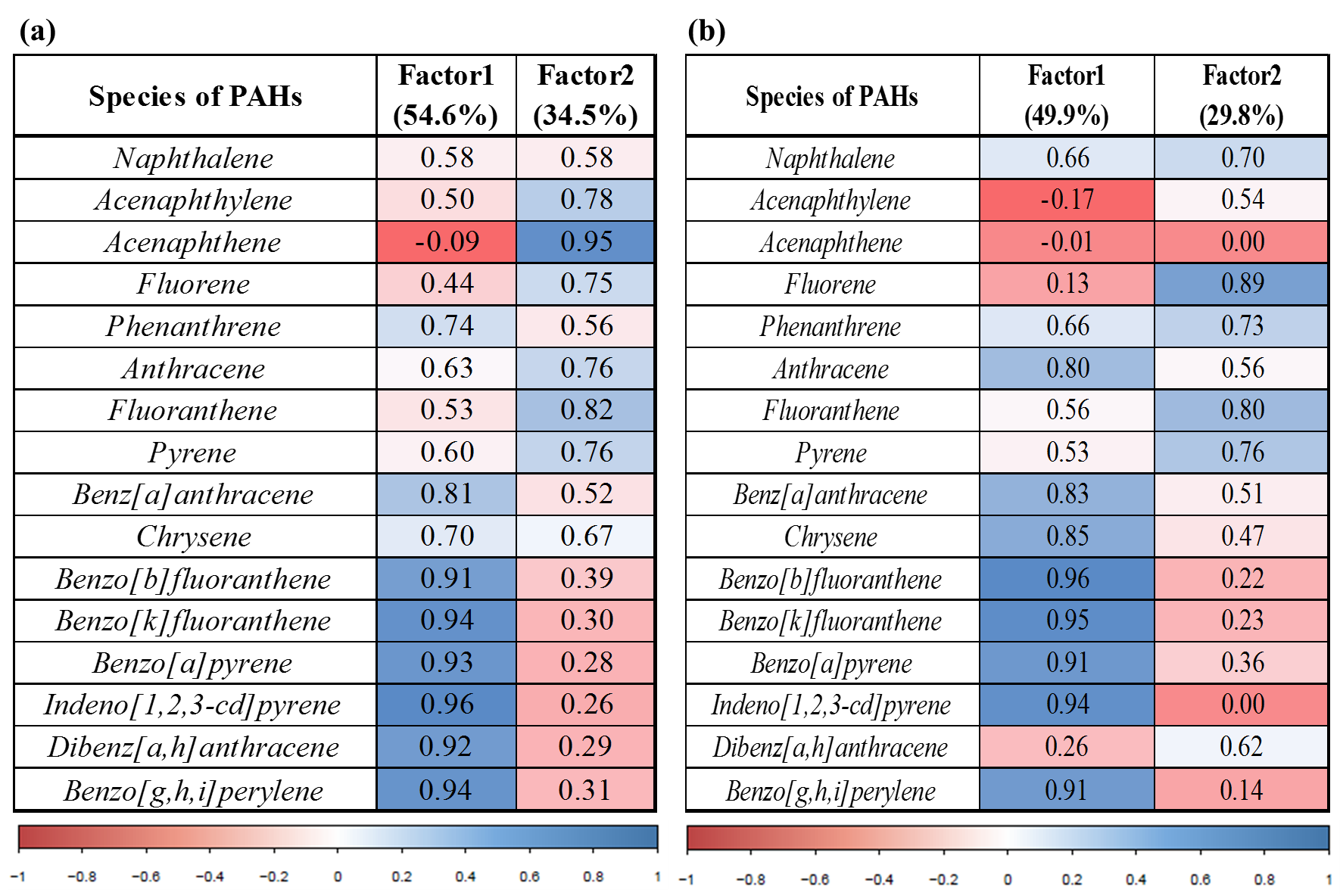


Table S5. Σ16BaP-TEQ andΣ16BaP-MEQ levels and excess cancer risks as a result of possible emission sources.

| Level (ng/m^3^) | Factor 1  Long-range transport | Factor 2  Gasoline vehicles | Factor 3  Coal combustion power plants | Factor 4  Diesel vehicles |
| --- | --- | --- | --- | --- |
| Σ16BaP-TEQ | 0.027 | 0.028 | 0.019 | 0.00019 |
| ECR | 2.37E-06 | 2.42E-06 | 1.68E-06 | 1.68E-08 |
| Σ8BaP-MEQ | 0.017 | 0.023 | 0.046 | 0.00084 |
| ECR | 1.46E-06 | 1.97E-06 | 4.02E-06 | 7.31E-08 |


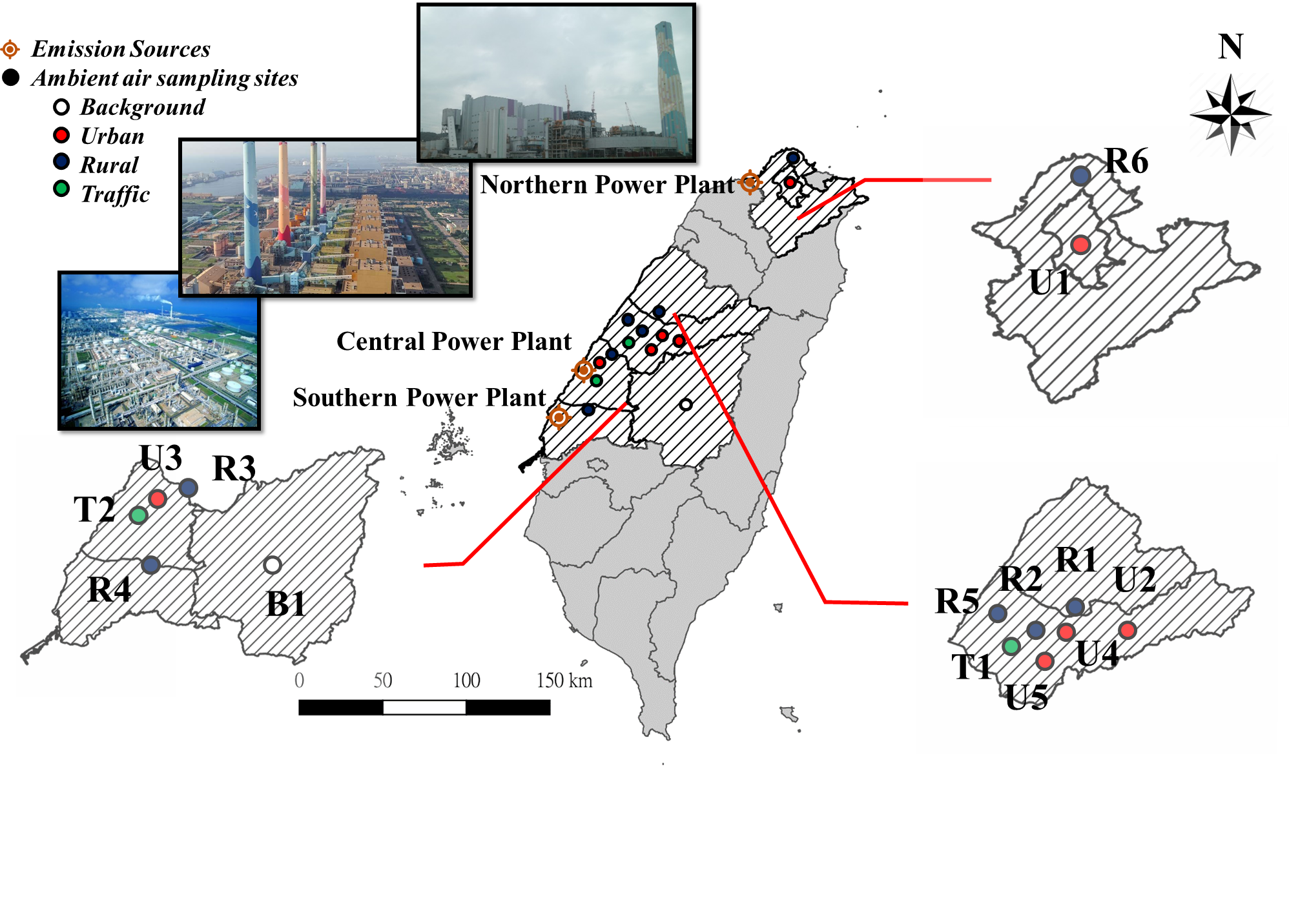


Figure S1 Map of emission sources and ambient air sampling sites.


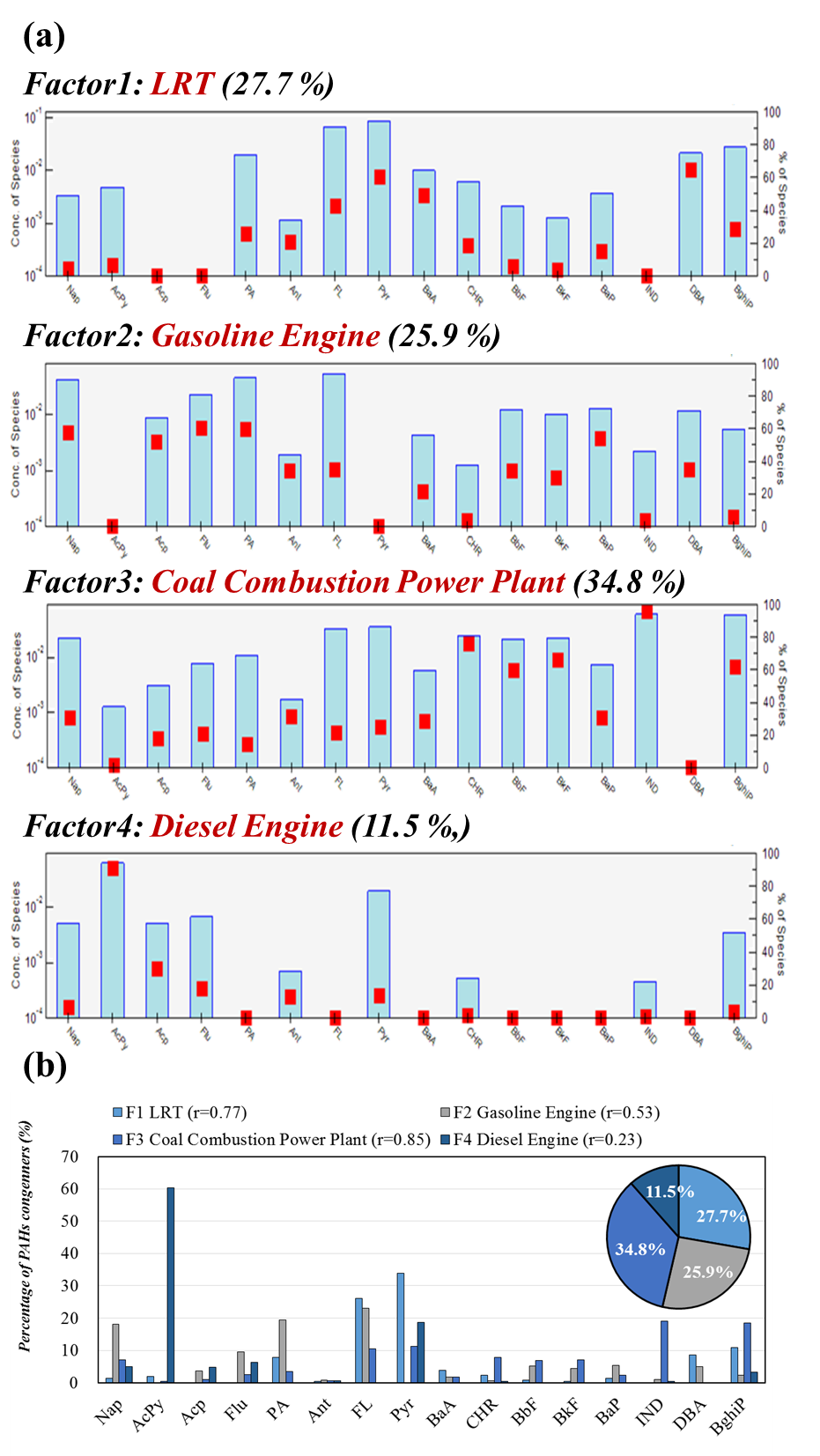


Figure S2 Sources profile of PMF in ambient air sampling sites (a) concentration of species in PAHs and (b) percentage of congeners in PAHs (n=66).


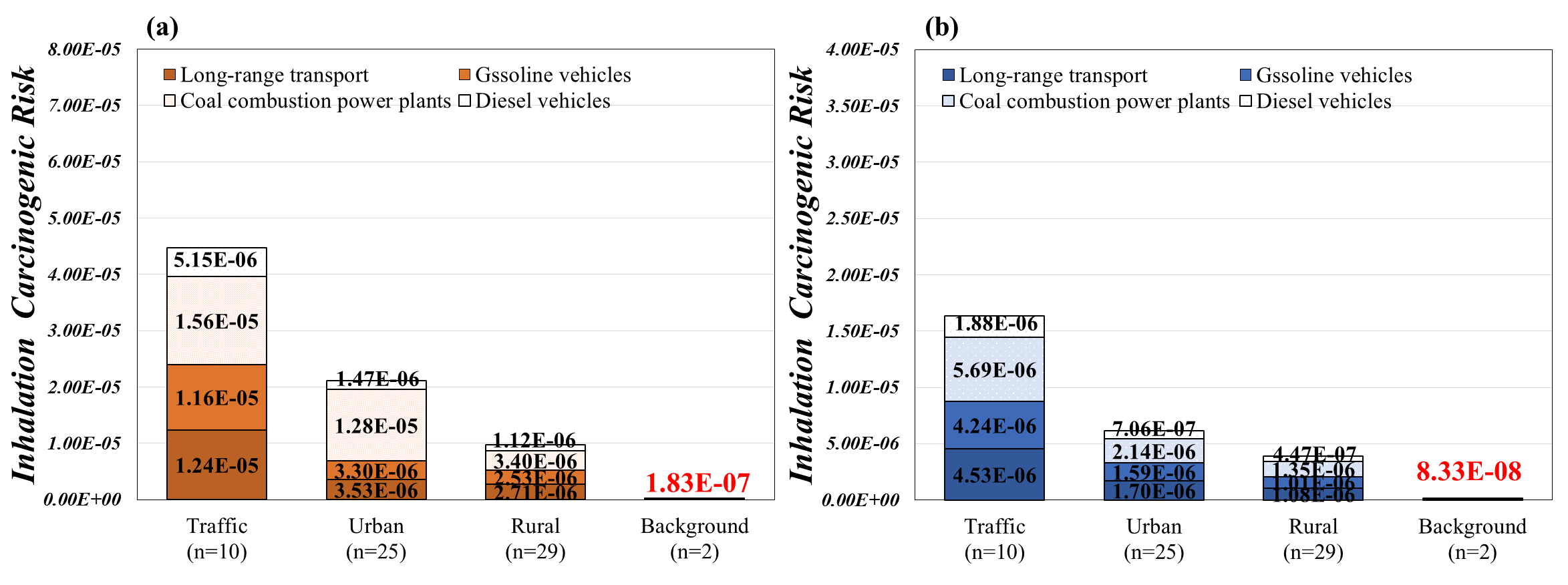


Figure S3 The ILCR was calculated based on (a) BaP-TEQ and (b) BaP-MEQ concentrations in possible contribution sources from different ambient air sampling sites.


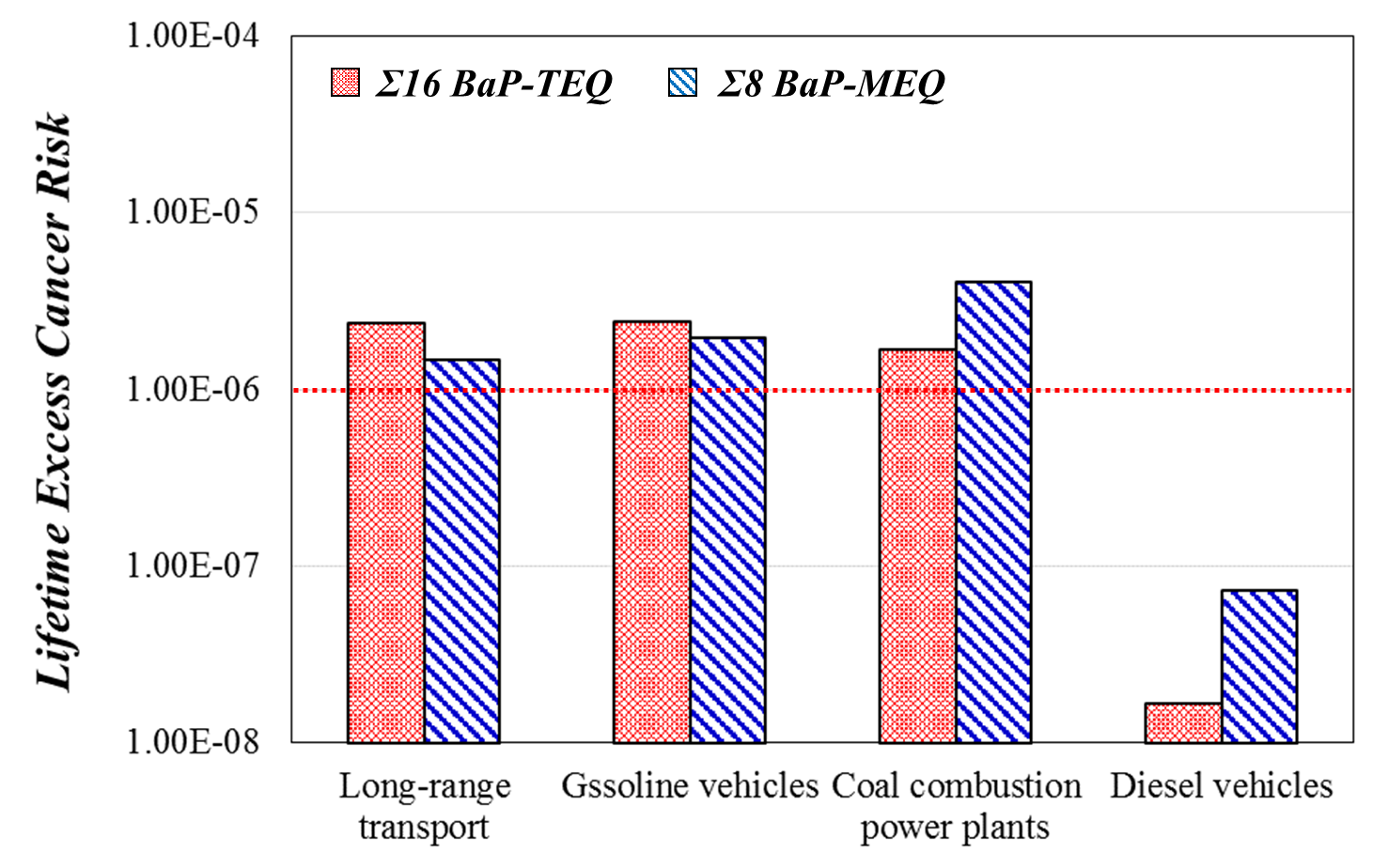


Figure S4 The ILCR was calculated from the result of PMF.
